# Conspecific Plasticity and Invasion: Invasive Populations of Chinese Tallow (*Triadica sebifera)* Have Performance Advantage over Native Populations Only in Low Soil Salinity

**DOI:** 10.1371/journal.pone.0074961

**Published:** 2013-09-05

**Authors:** Leiyi Chen, Candice J. Tiu, Shaolin Peng, Evan Siemann

**Affiliations:** 1 State Key Laboratory of Biocontrol, School of Life Sciences, Sun Yat-sen University, Guangzhou, Guangdong, China; 2 Department of Ecology and Evolutionary Biology, Rice University, Houston, Texas, United States of America; 3 State Key Laboratory of Vegetation and Environmental Change, Institute of Botany, Chinese Academy of Sciences, Beijing, China; 4 Department of Biological and Environmental Sciences, University of Tennessee at Chattanooga, Chattanooga, Tennessee, United States of America; Beijing Forestry University, China

## Abstract

Global climate change may increase biological invasions in part because invasive species may have greater phenotypic plasticity than native species. This may be especially important for abiotic stresses such as salt inundation related to increased hurricane activity or sea level rise. If invasive species indeed have greater plasticity, this may reflect genetic differences between populations in the native and introduced ranges. Here, we examined plasticity of functional and fitness-related traits of Chinese tallow (*Triadica sebifera*) populations from the introduced and native ranges that were grown along a gradient of soil salinity (control: 0 ppt; Low: 5 ppt; Medium: 10 ppt; High: 15 ppt) in a greenhouse. We used both norm reaction and plasticity index (PI_v_) to estimate the conspecific phenotypic plasticity variation between invasive and native populations. Overall, invasive populations had higher phenotypic plasticity of height growth rate (HGR), aboveground biomass, stem biomass and specific leaf area (SLA). The plasticity Index (PI_v_) of height growth rate (HGR) and SLA each were higher for plants from invasive populations. Absolute performance was always comparable or greater for plants from invasive populations versus native populations with the greatest differences at low stress levels. Our results were consistent with the “Master-of-some” pattern for invasive plants in which the fitness of introduced populations was greater in more benign conditions. This suggests that the greater conspecific phenotypic plasticity of invasive populations compared to native populations may increase invasion success in benign conditions but would not provide a potential interspecific competitive advantage in higher salinity soils that may occur with global climate change in coastal areas.

## Introduction

Extreme climate events, such as hurricanes, are predicted to be more frequent in the future and associated negative impacts on coastal ecosystems have gained increasing attention [1]. In addition to creating waterlogged and anoxic soils, hurricanes will increase electrolyte concentrations and osmotic potential of the soil solution and create a stressful, high salinity environment for both native and invasive plants [2]–[5]. Phenotypic plasticity, the potential of specific traits of a genotype to be expressed differently in distinct environments, is one of the mechanisms by which invasive plants can tolerate wide environmental variation and obtain an advantage in changing environments [6].

It has been shown that phenotypic plasticity of an individual or genotype may be adaptive, maladaptive or neutral with regard to an individual’s fitness. Only adaptive plasticity would contribute to invasion success [7]. Therefore, salinity stress may magnify, reduce, or have no effect on invasion success depending on the pattern of an invader’s phenotypic plasticity. For instance, high salinity could increase the relative competitive ability of the invasive grass *Bromus diandrus* and then might increase invasion intensity in coastal prairie at Bodega Head [8]. Plasticity in salt tolerance traits also allows invasive Japanese knotweed (*Fallopia japonica*) to live in saline habitats without a fixed adaptation to tolerate salt [9]. In contrast, native salt-adapted vegetation can be protected from salt-sensitive exotics by increasing soil salinity in salt marshes when the exotics have relative low fitness in salinity stress [10].

Studies that examine the role of salinity stress in exotic invasion, however, only focus on cross-species comparisons rather than conspecific comparisons between invasive and native genotypes. Rapid evolutionary change in the introduced range may be particularly important for invasive species because they often involve drastic changes in the invaded region [11]. While there is increasing evidence for genetic change in invasive plants in terms of growth, competitive ability, and herbivore defense [11]–[13], evolutionary change in phenotypic plasticity for conspecific invasive species has received much less attention [14], [15], especially in the context of soil salinity stress.

Chinese tallow tree (*Triadica sebifera*), an invasive species in the southeastern US, was found to increase its dominance in bottomland hardwood forest after Hurricane Katrina at the expense of less flood-tolerant or salt-tolerant native species [16], [17]. Hurricanes potentially create opportunities for tallow tree invasions both by removing existing vegetation and by creating high soil salinity conditions that native plants are less able to tolerate [18]. However, tallow tree is both naturally occurring and cultivated for 14 centuries in its native range but is not found in saline soils [19]. This niche difference of tallow tree between its native and introduced range suggests that there may be genetic variation in plasticity to abiotic stress (salinity, waterlogging, or anaerobic environment) between native and invasive populations of tallow.

In this study, we conducted a greenhouse experiment to compare the plasticity of morphological traits, leaf parameters and fitness-related traits of different tallow populations from the native and introduced ranges in the context of salt inundation. Specifically, we asked: 1) Do invasive populations have higher phenotypic plasticity to salt stress relative to native populations? 2) Do invasive populations have higher salt tolerance (fitness) relative to native populations?

## Materials and Methods

### Focal species

Chinese tallow tree is an invasive species in a variety of ecosystems throughout the southeastern United States [12], [20]–[23]. Previous studies suggested that tallow has evolved to be a faster-growing and less herbivore-resistant plant in its introduced range [12], [13], [24]–[26]. A recent study indicated that invasive populations of tallow had higher phenotypic plasticity of biomass than native populations did in response to variation in light conditions, but had similar plasticity across water stress conditions [27]. Compared to the native red maple (*Acer rubrum*), redbay (*Persea borbonia*), and baldcypress (*Taxodium distichum*), a single coastal South Carolina population of Chinese tallow tree survived longer at higher salinities, and showed higher tolerance to salinity [3], [28].

### Seed collection

In November and December 2010, we hand collected seeds from naturalized tallow trees in Texas (TX), Georgia (GA), Florida (FL), Louisiana (LA), USA, and from southern [Guangxi (GB), Guangdong (GL)] and northern [(Shanghai (SH), Hubei (HY), Zhejiang (ZH), Jiangxi (JX)] populations of tallow trees in China (Table 1). In each population we collected seeds from 4 to 15 trees. The geographic extent of populations ranged from hundreds of square meters to hundreds of hectares. Trees were meters to tens of meters away from the nearest tree from which seeds were collected. All seed collections were from public areas where no permission was required for collection. Tallow tree is not an endangered or protected species in either country. The collection in the US included populations descended from the original introduction into Savannah, GA [GA population] from southern China and those from a later introduction throughout the Gulf Coast from populations in the north-east part of the range [closest population here is Shanghai] [29], [30]. Seeds were air-dried and stored in the refrigerator at 4°C in the dark after collecting. Before seeds were planted, we removed the waxy seed coats unrelated to seed provisioning by soaking seeds in water and detergent. Two hundred randomly selected seeds from each population were individually sown in March 2011 in 65 ml conical containers (Stuewe & Sons, Corvallis, OR, USA) with commercial soil and placed in a greenhouse at Rice University, Houston, TX, USA. Sixteen seedlings from each population were bare root transplanted into pots (3.5 liters) containing topsoil taken from Justin Hurst Wildlife Management Area (Jones Creek, TX, USA) in June 2011 (soil was Pledger clay – a very-fine, smectitic, hyperthermic Typic Hapludert) in a randomized design. Justin Hurst Wildlife Management Area is a grassland area on the coast, invaded by tallow and vulnerable to storm surge, especially with sea level rise. Soil was collected from a recently tilled fire break. To minimize maternal effects due to differences in seed qualities, seedlings of similar size were selected for the experiment. We measured stem height and recorded the number of leaves for all seedlings before transplanting. Height (origin: F_1,10_ = 0.33, *P* = 0.58, saline:F_3,174_ = 0.69, *P* = 0.60, origin x salinity: F_3,30_ = 0.62, *P* = 0.61) and number of leaves (origin: F_1,10_ = 0.07, *P* = 0.80, saline:F_3,174_ = 1.03, *P* = 0.38, origin x salinity: F_3,30_ = 0.23, *P* = 0.87) were independent of treatments when transplanted.

**Table 1:** Native (China) and invasive (US) populations of tallow that were used in this study.

Origin	Population	Location	Latitude	Longitude
**China**	SH	Shanghai	31^o^ 31’ N	121^o^ 51’ E
	HY	Yingshan, Hubei Province	30^o^ 46’ N	115^o^ 36’ E
	GB	Baihuahu, Guizhou Province	26^ o^ 41’ N	106^ o^ 31’ E
	GL	Lianzhou, Guangdong Province	24^ o^ 47’ N	112^ o^ 23’ E
	ZH	Hangzhou, Zhejiang Province	30^ o^ 16’ N	120^ o^ 9’ E
	JY	Yingtan, Jiangxi Province	28^ o^ 16’ N	117^ o^ 41’ E
**USA**	GA-1	Hutchinson Island, GA	32^ o^ 60’ N	81^ o^ 6’ W
	TX-1	Houston, TX	29^ o^ 42’ N	95^ o^ 25’ W
	LA-5	Pumpkin Center, LA	30^ o^ 29’ N	90^ o^ 33’ W
	LA-4	Baton Rouge, LA	30^ o^ 23’ N	91^ o^ 10’ W
	LA-1	Lake Charles, LA	30^ o^ 14’ N	93^ o^ 10’ W
	FL-3	Callahan, FL	30^ o^ 35’ N	81^ o^ 48’ W

### Experimental design

The experiment was carried out between June and September 2011 in a greenhouse at Rice University, Houston, TX, USA. We used a factorial design that consisted of 2 origins (US and China), 6 populations and a 4-level saline stress treatment with four replicates (2×6×4×4 = 192 pots). All the pots had one individual plant and were arranged in a complete randomized design in June 2011. This study examined genotype-level plasticity, which is the capacity of genotypes (here expressed as populations from same origin) to produce different phenotypes across different levels of salinity stress. In the saline stress treatment, four concentrations of salt were applied daily (Control: 0 ppt water; Low: 5 ppt water; Medium: 10 ppt water; High: 15 ppt water). It has reported that a 3 meter storm surge, could bring large volumes of water inland with salinity up to 20 parts per thousand (ppt) [31], [32]. We used commercial sea salt powder for making artificial seawater (Natural Sea Salt Mix, Oceanic Systems, Inc., Franklin, WI, USA) which included elements in the same ratios as natural seawater. Seedlings were watered with tap water for one week to minimize transplant shock and seedling mortality before the experiment began.

### Soil salinity

Soil salinity and conductivity were measured at the end of the experiment in a subset of pots representing six to nine pots of each salinity treatment. Soil was collected, dried, weighed, ground, and then distilled water was added. We measured salinity and conductivity in the water with a conductivity meter (Extech instruments ExStik EC400).

### Leaf parameters

Two leaf traits related to plant performance and fitness were calculated: specific leaf area (SLA; leaf area per unit leaf mass, cm^2^/g) and leaf area ratio (LAR, total leaf area per whole plant mass, cm^2^/g). Leaf parameters were sampled at the time of harvest. Eight to ten leaves of each seedling were randomly selected to measure the leaf area immediately after harvesting. We scanned the leaves to measure the leaf area using IMAGE J software (National Institute of Health, Bethesda, MA). Finally, we measured the scanned leaf dry weight after drying them at 60°C for 96h. We calculated the SLA and the LAR as follows: SLA = leaf area/leaf mass; LAR = SLA * leaf mass ratio (LMR; leaf mass per whole plant mass).

### Morphological traits

Stem height and number of leaves were measured every week during the experiment and at harvest. Based on stem height measurements, height growth rate [HGR =  (final height-initial height)/initial height] was calculated [33]. In August 2011, 85 days from the start of the experiment, we harvested all the plants. Leaves (including petioles) were clipped from the stems and branches. Plants were clipped at the soil base (leaves and stems were then separated) and roots were gently washed from the soil. After measurements for SLA and LAR, plant samples were dried at 60°C for 96 hours and weighed to determine root biomass, stem biomass, and leaf biomass. Three variables were calculated from these measurements: root to shoot ratio (R/S), leaf mass ratio (LMR; leaf mass per whole plant mass), and leaf area ratio (LAR; total leaf area per whole plant mass)

### Fitness-related traits

We used survival time and final biomass as fitness surrogates. Survival was monitored from June 2011 every week until the end of the experiment. Seedlings were considered dead only when the stem withered and died. Salt tolerance has usually been assessed as the percentage final biomass production in saline versus control conditions over a prolonged period of time [34], [35].

### Estimators of phenotypic plasticity

In this study, we used both reaction norm and quantitative estimator (plasticity indices) to estimate the phenotypic plasticity of invasive and native populations of Chinese tallow tree under salinity stress. A reaction norm is the range of values of a trait, expressed over a range of environments [9] and it is the most immediate way of exploring phenotypic plasticity [36], [37]. It is indicated by a significant statistical interaction between environment and genetic origin. In addition, using plasticity indices as quantitative estimation of the phenotypic change induced by the environments is a crucial step in ecological approaches to phenotypic plasticity [37]. Here we generated a Phenotypic Plasticity Index (PI_v_) for each trait [6]. The index ranges from zero (no plasticity) to one (maximum plasticity) and is the difference between the minimum and maximum value of the treatment means of a trait divided by the maximum [38]. We calculated the PI_v_ for each population and PI_v_ for population origins was calculated using average values for populations.

### Statistical analysis

To compare plant growth and plasticity of invasive populations and native populations, we conducted mixed ANOVAS (Proc MIXED, SAS 9.0). The models included origin (native vs. invasive), saline (control, low, medium, high), and their interaction as fixed effects, and population nested within origin as a random effect. We used the population (origin) and population × saline (origin) terms as the error terms for significance tests of origin and origin × saline, respectively. When a significant effect was detected for an interaction term, further LSD multiple comparison tests for differences among treatments were made using follow up analyses that included only a subset of the data. Following the recommendations of Moran (2003), sequential Bonferroni corrections for multiple statistical tests were not conducted [39]. As recommended by Moran (2003), all P-values are reported [39]. To compare the differences in PI_V_ between native and invasive populations for a given trait, we conducted a Wilcoxon rank sum test. Significant effects of origin on performance of tallow in the common environment would indicate genetic differences in plant traits between native (China) and introduced (U.S.) populations [40]. Significant interactions between origin and treatment would indicate genetic differences in plasticity between native and introduced populations [41].

We conducted an additional analysis of survival which used the percent of seedlings surviving within a population as the response variable. This ANOVA only had origin, saline, and their interaction as predictors.

## Results

### Saline stress effect

**Figure 1:**
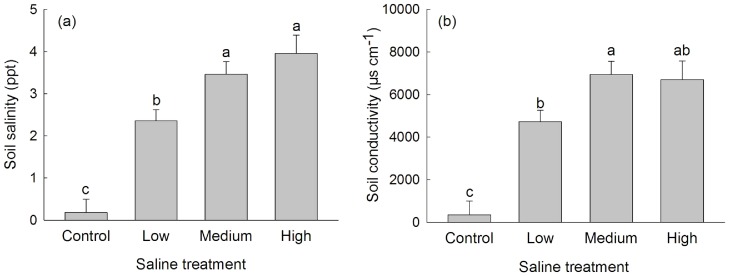
Soil salinity and conductivity in different saline stress treatments. Values are means + 1 SE. Means with the same letters were not significantly different in post-hoc multiple comparisons of means at the *P* = 0.05 level.

Soil conductivity (*F*_3,14_ = 21.01, *P*<0.0001) and soil salinity (*F*_3,14_ = 24.98, *P*<0.0001) depended on saline treatment and were significantly higher in pots in saline stress treatments than in control pots (Fig. 1). Soil conductivity and soil salinity were independent of origin (*F*_1,10_ = 0.04, *P* = 0.85; *F*_1,10_ = 0.61; *P* = 0.45).

### Differences between invasive and native populations in conspecific plasticity

**Figure 2:**
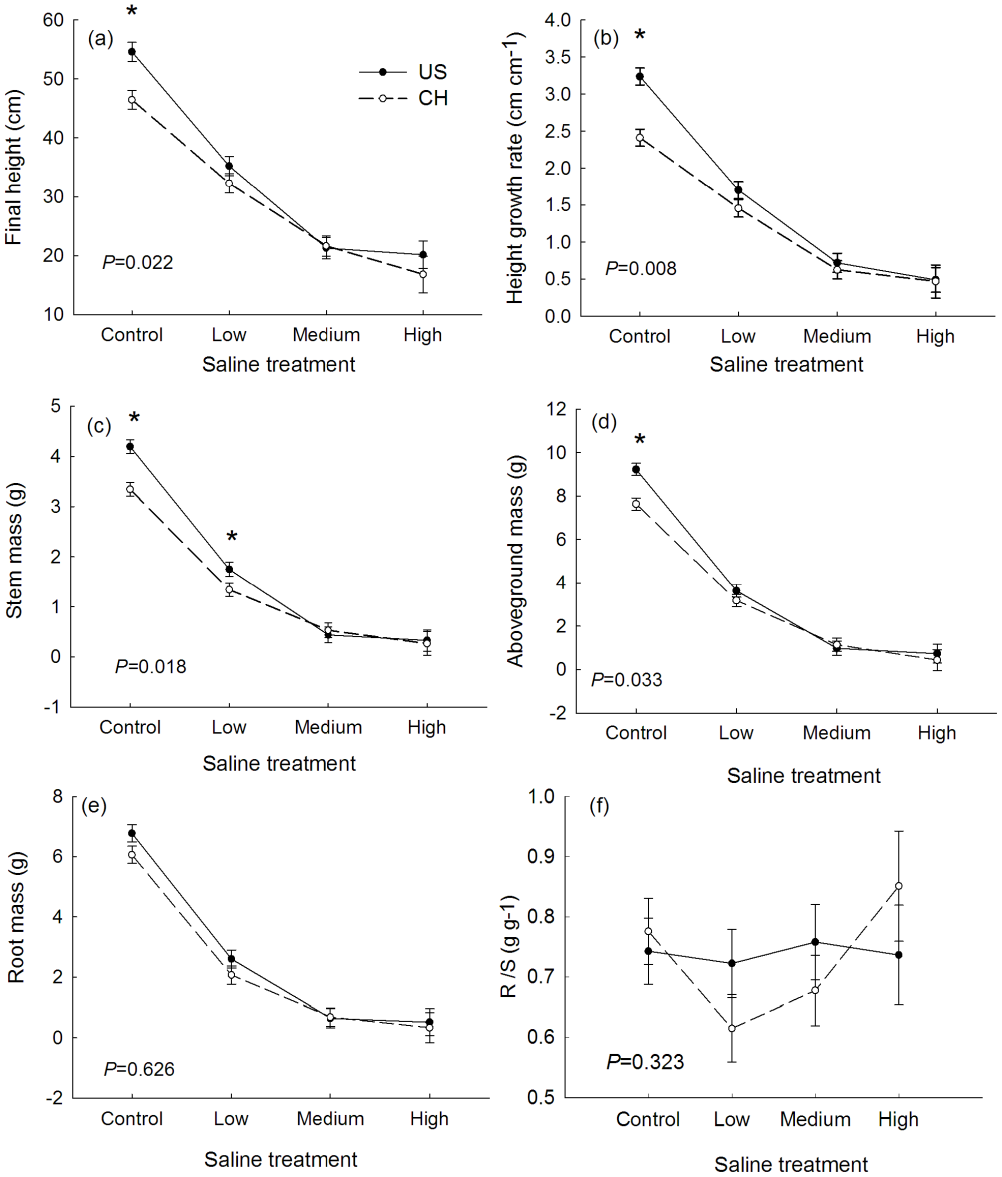
Functional traits of invasive and native population of tallow in different saline stress treatments. (a) Final height, (b) height growth rate, (c) stem mass, (d) aboveground mass (e) root mass, and (f) root to shoot ratio. Values are means ± 1 SE. P-values are the significance of the interaction effect between origin×saline (full ANOVA results are in Table 2). Asterisk (*) indicates significant differences between invasive and native populations in a saline stress treatment at the 0.05 level according to post-hoc multiple comparisons of means in ANOVA.

**Figure 3:**
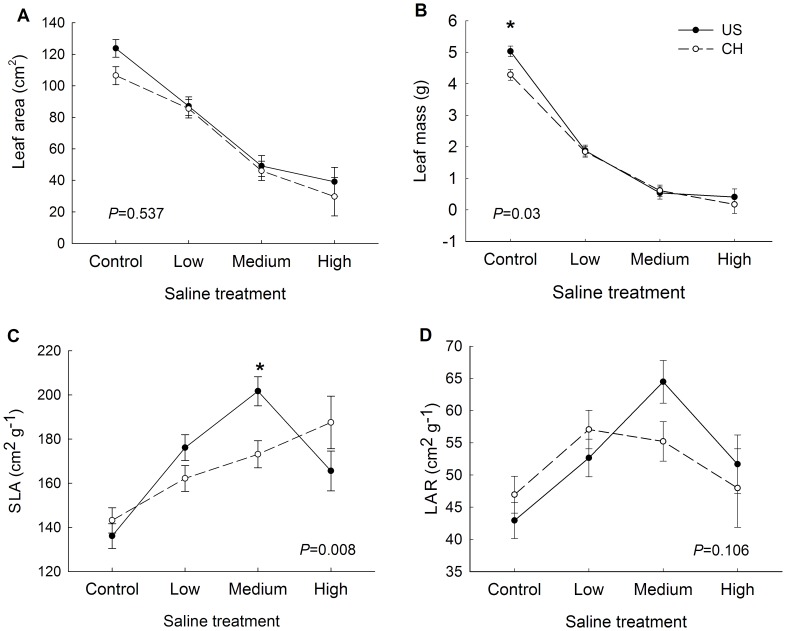
Leaf functional traits of invasive and native population of tallow at different saline stress treatments. (a) Leaf area, (b) leaf mass, (c) SLA and (d) LAR. Values are means ± 1 SE. P-values are the significance of the interaction effect between origin×saline (full ANOVA results are in Table 2). Asterisk (*) indicates significant differences between invasive and native populations in a saline stress treatment at the 0.05 level according to post-hoc multiple comparisons of means in ANOVA.

In general, tallow had a lower height growth rate, smaller leaf area and less leaf, stem and root biomass and higher SLA and LAR (Table 2; Fig. 2; Fig. 3) in saline stress conditions than those of plants in control pots. However, final height, height growth rate, stem mass, and aboveground biomass of invasive populations were only higher than that of the native populations in the no saline stress condition (control pots; Fig. 2). There were no differences in height growth and stem, leaf, and root biomass between invasive and native populations in any salt stress treatment (Fig. 2). For the leaf traits, invasive populations had higher SLA in medium saline stress than that of native populations (Fig. 3).

**Table 2:** ANOVAs for the effects of saline stress on plant- and leaf-level traits and fitness related traits of seedlings from native and invasive populations of tallow, significant results are shown in bold.

	Origin			saline			Origin×saline		
	*df*	*F*	*P*	*df*	*F*	*P*	*df*	*F*	*P*
**Morphological traits**									
Final height	1,10	1.12	0.315	3,132	274.60	**<0.001**	3,30	3.73	**0.022**
Height growth rate	1,10	5.62	**0.036**	3,132	177.65	**<0.001**	3,30	4.73	**0.008**
Stem mass	1,10	6.72	**0.030**	3,132	219.34	**<0.001**	3,30	3.90	**0.018**
Aboveground mass	1,10	5.03	**0.049**	3,132	265.71	**<0.001**	3,30	3.32	**0.033**
Root mass	1,10	1.93	0.195	3,132	153.92	**<0.001**	3,30	0.59	0.626
R/S	1,10	0.04	0.850	3,132	1.48	0.223	3,30	1.21	0.323
Leaf mass	1,10	1.91	0.200	3,132	178.42	**<0.001**	3,30	1.24	0.313
Leaf area	1,10	2.19	0.1698	3,124	55.27	**<0.001**	3,30	0.74	0.537
**Leaf parameters**									
SLA	1,10	0.36	0.564	3,124	24.77	**<0.001**	3,30	4.73	**0.008**
LAR	1,10	0.18	0.680	3,124	9.00	**<0.001**	3,30	2.22	0.106
**Fitness-related traits**									
Survival time	1,10	0.02	0.905	3,174	31.45	**<0.001**	3,30	0.66	0.583
Total biomass	1,10	5.02	**0.049**	3,132	247.05	**<0.001**	3,30	1.86	0.158

Three morphological traits (height growth rate, aboveground biomass, stem biomass) and one leaf trait (SLA) showed a conspecific plasticity difference between invasive and native populations indicated by a significant saline × origin interaction (Table 2). In general, plants from populations in both ranges showed no differences in the magnitude of plasticity in root to shoot ratio (Table 2). This reaction norm indicated that the fitness of the invasive populations was greater in the more benign conditions (Fig. 2). Similar results were found in the phenotypic plasticity index (PI_v_). The PI_v_ of height growth rate and SLA of invasive populations is higher than that of native populations (Table 3). For example, the height growth rate of invasive populations, on average, increased 85% in benign conditions. In contrast, native populations increased 81% when they were in benign condition (Fig. 2B).

**Table 3.Plasticity:** indexes (PI_v_) of invasive and native populations of tallow for different functional traits, significant results between populations are shown in bold.

	Functional traits								
Origin	Height	HGR	Root mass	Stem mass	Leaf mass	Aboveground mass	R/S	SLA	LAR
US	0.65	**0.85**	0.93	0.92	0.93	0.93	0.27	**0.32**	0.34
CH	0.62	**0.81**	0.94	0.92	0.96	0.94	0.36	**0.23**	0.36

### Differences between invasive and native populations in fitness-related traits

**Figure 4:**
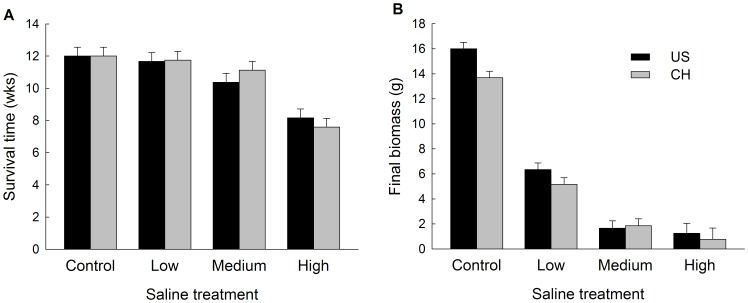
Fitness related traits of invasive and native populations of tallow at different saline stress conditions. (a) Survival time and (b) final biomass. Values are means + 1 SE.

Survival time of seedlings of both origins (i.e. native and invasive populations) decreased significantly with increasing saline concentration (Table 2; Fig. 4A). Survival of invasive and native populations were affected in a similar way by saline water as survival of plants did not differ between native and invasive populations in each salt stress condition (Table 2). Average survival of populations depended on salinity (F_1,30_ = 45.00, P<0.001) but not population origin (F_1,10_ = 0.08, P = 0.781) or their interaction (F_3,30_ = 1.07, P = 0.378).

Invasive populations had higher total biomass than native populations (Table 2). However, total biomass decreased significantly with increasing salinity but did not depend on origin or the interaction of origin and salinity (Fig. 4B). Biomass did not show a plasticity difference between the two origins in response to saline stress conditions (Table 2), as indicated by no significant effect of saline treatment × origin.

## Discussion

Higher abiotic stress tolerance of invasive species compared to native species, which could contribute to invasion success under stressful conditions, can result from intrinsic attributes of the invader (pre-adaption) or evolution of phenotypic plasticity after introduction to a novel environment [42]–[44]. Only a few studies have evaluated conspecific phenotypic plasticity differences between invasive and native populations of an exotic invader in a wide range of conditions, from favorable to stressful conditions [14], [27], [29], [45]. In this study, we found that differences in plasticity between origins varied among traits. Overall, plasticity of four morphological traits (final height, HGR, aboveground biomass and stem mass) and one leaf trait (SLA) were higher for invasive tallow populations compared to native populations, especially for plasticity of HGR and SLA.

Growth rate is a key trait for maintenance of fitness when plants grow in stressful environments because survival and reproduction depend on plant size [46]. Rapid height growth rate (HGR) is one of the most particularly important traits of invasive species [47]–[49] and such a life-history pattern has been found in many other successful invaders. For instance, high seedling RGR under optimal conditions was the most important trait associated with invasiveness for pine species [50]. Similar results were found recently from near maximum RGR studies of some herbaceous plants under optimal conditions [51]–[53]. In the present study, HGR of invasive populations of tallow was about 34.3% higher than that of native populations when the environmental was benign (i.e. no salinity, Fig. 2). In addition, stem mass of invasive populations was also significantly higher than that of native populations only when the environmental was benign or with low saline stress (5 ppt). Thus the apparent unusual high salinity tolerance of tallow compared to native species in coastal forests in its introduced range is more consistent with intrinsic attribute.

Negative effects of abiotic stress on plant performance may also be limited by phenotypically plastic allocation patterns. Despite the great reduction in leaf area when saline concentrations increased (Fig. 3), which is the first symptom of salt stress in the plants to keep the content of moisture [54], [55], SLA of invasive populations in medium saline water remained high compared to native populations of tallow in our results. Although higher SLA in high resource environments may allow the invasive species to grow quickly [56]–[58], higher transpiration of plants will be increased by higher SLA under high saline or water stress. Therefore, invasive populations with high SLA in saline stress did not reflect an adaptive response.

Invasive populations outperformed native populations when the environment was benign in terms of morphological traits, leaf traits and fitness-related traits. The consistency of differences between invasive and native populations across the different traits in favorable resource conditions could reflect that many traits vary in concert because some traits are closely related [50], [59], [60]; for instance, specific leaf area and stem mass are frequently positively associated with high relative growth rates [61]. High growth rates in turn are likely to result in high competitive ability and total biomass production (fitness-related trait). Other experiments consistently indicate that favorable environmental resources (e.g. increased soil nutrient availability) would promote the invasion of tallow [62]–[68]. However, in stressful conditions, invasive populations of tallow performed comparably to native populations, which indicated that high salinity tolerance found in tallow tree compared to native trees in US may be an intrinsic attribute of tallow and not a trait novel to invasive populations. In general, stressful environments have lower invasibility than habitats with abundant available resources; for example, salt marshes are rarely invaded by non-natives due to their hyper-saline soil [69]. Similar performance of invasive and native populations in saline conditions supports the idea that trait plasticity is not always constrained in low resource environments and plasticity may be costly in stressful environments [6], [38].

Two belowground effects mediate plant biomass reduction in increasing soil salinity conditions: osmotic effects from declining soil solute potential that create water stress, and ionic effects due to seed or seedling ion uptake and/or accumulation [70]. It has also been assumed that belowground resource capture ability is size dependent [71], [72]. Root system size and biomass allocation may positively associated with belowground competition for resources (e.g. [72]–[74]), which may result in different tolerances in saline stressful conditions. As a result, the growth consequence of belowground stress in the context of hurricane and sea level rise may be closely related to root traits, which may be the most important in determining vulnerability to elevated soil salinity [75]. In our study, conspecific invasive and native populations of tallow had similar root biomass allocation and did not show differences in phenotypic plasticity. In contrast, aboveground traits (i.e. stem mass, height growth rate) of tallow had significant differences and showed differences in phenotypic plasticity along a salinity gradient. This similar root trait of invasive and native populations may drive similar performance when belowground stress increases but a large difference in aboveground traits may drive greater performance of invasive population in conditions with low belowground stress and abundant resources.

Two strategies critical to the invasiveness of exotic species are stress tolerance and resource opportunism [76]. More generally, increased tolerance to abiotic stress is believed to trade off against growth and competitive abilities in plants as a result of resource limitations that drive the evolution of allocation strategies [77]. Accordingly, phenotypic plasticity could contribute to invasion success in following three scenarios: a) Phenotypic plasticity in response to stressful conditions enables greater fitness homoeostasis (“Jack-of-all-trades”). b) Phenotypic plasticity in response to more favorable conditions enables a large increase in fitness (“Master-of-some”). (c) A species with both attributes of robustness in the face of stress and opportunistic in the face of benign conditions (“Jack-and-master”) [11], [78]. Following this prediction, populations of tallow of different origins followed a “Master-of-some” strategy in stressful saline conditions. Plant populations can have different mechanisms to achieve invasion success depending on which resource gradients are being considered. Tallow was found to follow a “Master-of-some” strategy in a soil water gradient but a “Jack-of-all-trades” along a light gradient when competing with native grasses [27]. But it follows a “Master-of-some” strategy along a light gradient when competing with native trees in floodplain forests in the US in which tallow was outperformed by the most shade-tolerant natives in the lowest light conditions. However, tallow has increasing performance advantages over all natives for increasing light levels above its compensation point [79], [80]. Only some vigorous invaders possess both high stress tolerance and higher resource capture ability [81]. For instance, Godoy et al. (2011) found invasive species had higher fitness in both high light availability and shade stress condition [82]. Invasive populations of *Phragmites australis* was also found to have jack-and master phenotypic plasticity when facing imminent global change conditions [83].

Plasticity of functional traits is unlikely to affect invasiveness unless that plasticity contributes positively to fitness [11], [77], [84], [85]. Thus only adaptive plasticity will be advantageous [7], [86]. In our study, invasive populations did not show greater fitness in saline stress conditions. The fitness of the introduced populations was greater only in the more benign conditions and they responded just as well as native populations when saline stress is increased. Non-adaptive plasticity of plants is frequently found in hostile environments [87], [88]. Sea water carried by the storm surge of a hurricane will significantly increase the soil salinity level, giving a highly stressful condition to coastal ecosystems [3], [89]. Schumacher et al. (2008) pointed out that high phenotypic plasticity of some fast-growing invasive species did not contribute to their fitness when both light and water resources are limited [90]. Higher plasticity in water use efficiency (WUE), but less drought tolerance was also found in invasive dandelions [91].

Tallow does not occur in coastal areas in its native range even though our results indicated that native and invasive populations have comparable soil salinity tolerances in the introduced range. That difference in distribution area might result from different biotic interactions in the native range of tallow. Aboveground herbivore and belowground soil organisms could inhibit the growth of native population of tallow in the native range [92], [93]. Both herbivore and pathogen pressure might together inhibit the growth of tallow in saline conditions in the native range [94]. At the same time, rates of association with mycorrhizal fungi are smaller in the native range even after controlling for tallow population origin [93]. More generally, belowground interactions may limit tallow from coastal regions in the native range. Further studies focused on how enemy release might allow expansion of the species’ niche in the introduced range should be carried on. In addition, except for salinity stress, hurricanes and sea-level rise will have other effects on the physical environment, e.g. create a waterlogged and anoxic habitat, which could impact on growth and fitness of *Triadica* and other species in the introduced range. For example, it was reported that native trees were very susceptible to saltwater flooding, whereas tallow tree seedlings were the most tolerant and were able to survive up to 5 days of flooding [3]. Further conspecific phenotypic plasticity comparisons between different genotypes should investigate the interaction among multiple abiotic stresses that might be induced by hurricane and sea-level rise simultaneously.

In conclusion, our results suggested that invasive tallow populations only have higher phenotypic plasticity and fitness compared to native populations in benign conditions, but perform comparably versus native populations at high stress levels which followed the “Master-of-some” invasion pattern. The high salinity tolerance of tallow in the introduced range does not appear to be an evolved trait but rather seems to reflect pre-adaptation to those stressful conditions possibly in combination with unusual biotic interactions.
